# The Role of Spirometry and MMEF in Pediatric Asthma Monitoring and Prediction of Exacerbations

**DOI:** 10.3390/children12101398

**Published:** 2025-10-16

**Authors:** Paraschiva Chereches-Panta, Ioana Marica, Valentina Sas, Alina Petronela Bouari-Coblișan, Sorin Claudiu Man

**Affiliations:** 1 Third Pediatric Discipline, Mother and Child Department, Faculty of Medicine, Iuliu Hațieganu University of Medicine and Pharmacy, 400347 Cluj-Napoca, Romania; pusachereches@umfcluj.ro (P.C.-P.);; 2 Third Pediatric Clinic, Clinical Hospital for Pediatric Emergencies, 400315 Cluj-Napoca, Romania; petronela.coblisan@umfcluj.ro; 3 Clinic of Pediatric Psychiatry and Drug Addiction, Clinical Hospital for Pediatric Emergencies, 400489 Cluj-Napoca, Romania; marica.ioana@elearn.umfcluj.ro; 4 General Nursing Discipline, Faculty of Nursing and Health Sciences, Iuliu Hațieganu University of Medicine and Pharmacy, 400124 Cluj-Napoca, Romania

**Keywords:** asthma, children, exacerbations risk, small airways dysfunction, maximum mid-expiratory flow (MMEF)

## Abstract

**Background**: Asthma is the most common chronic disease during childhood. Spirometry is recommended as a reliable lung function test. Several studies have demonstrated the lack of use of spirometry for both diagnostic confirmation and monitoring. Using subjective symptom control tests alone may underestimate the risk for future asthma attacks. **Methods/Objectives**: We conducted a retrospective, observational study in a single pediatric centre in Romania. The main objectives of the study were to analyse the quality of spirometry in children and to emphasise the importance of performing accurate spirometry for asthma monitoring. The secondary objective was to evaluate if forced expiratory volume in the first second (FEV1) and mid-maximum expiratory flow (MMEF) values are predictive markers for future exacerbations in children with asthma. **Results**: The study group included 416 patients between 5 and 18 years who performed at least one spirometry. The success rate for spirometry in our study was 66.3%. In a subsequent study group of 88 patients we monitored spirometry initially and after 12 months. We found a statistically significant difference between FEV1 and MMEF in the controlled, partially controlled and uncontrolled groups (*p* = 0.0102 and *p* = 0.0001). Our study showed no association between FEV1 and risk for exacerbations (Rs = −0.156, *p* = 0.146) and an acceptably negative (Rs = −0.30) and statistically significant (*p* = 0.040) correlation between initial MMEF values and the number of exacerbations. **Conclusions**: Low initial MMEF values correlate with the number of exacerbations in a 12-month follow-up period. This suggests that evaluating MMEF alongside FEV1 in children with asthma could contribute to better identification of the risk of exacerbation.

## 1. Introduction

Asthma is a chronic respiratory disease characterised by persistent airway inflammation that causes bronchial hyper-responsiveness. Exposure to a wide range of factors triggers episodes of wheezing, cough, and shortness of breath. About 14% of children worldwide are diagnosed with asthma, making it the most common chronic respiratory disease in childhood [1].

The correct diagnosis and monitoring should be a priority. Current guidelines [1] recommend the following diagnostic criteria for asthma: a history of variable respiratory symptoms and confirmation of airflow variability. Pulmonary function tests can demonstrate airflow limitation and hyper-responsiveness through positive reversibility testing after bronchodilation, as well as via positive exercise challenge test and/or positive bronchial methacholine challenge test. Indicators of bronchial hyper-responsiveness include peak expiratory flow variability measured twice daily over two weeks and improved lung function following four weeks of anti-inflammatory treatment. Once asthma is diagnosed, pulmonologists play a central role in assessing severity, establishing the therapeutic step, and monitoring symptom control and exacerbation risk. After changing treatment, patients with controlled asthma should be seen every six months, while those with partially controlled or uncontrolled asthma should be reviewed approximately every three months [2]. During each follow-up, the assessment should include a pulmonary function test, an Asthma Control Test (ACT), and/or an Asthma Control Questionnaire completed by the patient [1].

Spirometry is currently the most commonly used and reliable lung function test for diagnosing asthma and monitoring its progression. Although it is a non-invasive and relatively simple test, accurate interpretation of spirometry depends on proper technique during the manoeuvre. In young children, most software includes animation programmes to improve performance during expiration. The American Thoracic Society (ATS) and European Respiratory Society (ERS) joint task force provides guidelines for spirometry interpretation [2,3]. Many young children have smaller absolute lung volumes and larger airway sizes. They cannot produce a forced expired volume in 1 s (FEV1) because they are unable to sustain forced expiration for more than 1 s [4]. Acceptability criteria are essential for appropriate spirometry interpretation. These include the following: a rapid rise to peak expiratory flow without evidence of leak, mouthpiece obstruction, glottis closure, or cough; smooth expiration to residual volume on flow-volume curves; and an appropriate plateau on the volume-time curve with no signs of any inspiration [4].

The FEV1 and the forced vital capacity (FVC) are used to classify airflow obstruction and its severity. They reflect the function of the larger airways. Maximal mid-expiratory flow (MMEF) is the mean flow rate between 25% and 75% of FVC, and is used to assess the function of small airways. Its decrease is a marker for small airways dysfunction (SAD) [5]. A value of <65% of predicted of any two of the following parameters: forced expiratory flow between 25 and 75% of FVC (FEF25-75), forced expiratory flow at 50% of FVC (FEF50), or forced expiratory flow at 75% of FVC is currently used for the definition of SAD [6]. The MMEF is measured during exhalation, independent of effort, on the flow-volume curve. Reduced MMEF suggests dynamic peripheral airway obstruction and appears as a scoop on the flow-volume curve, which can be difficult to interpret given the wide range of expected values [5]. Airway dysfunction is not evenly distributed throughout the respiratory tract. A multivariate analysis of lung function in disease control showed that although FEV1 does not appear to correlate with the level of control, a decrease in FEF50 has a significant role in poor asthma control [7]. After treatment, FEV1 improves in patients with uncontrolled asthma, while SAD may persist.

However, several studies have demonstrated the lack of use of spirometry for both diagnostic confirmation and monitoring [8]. Its importance is highlighted in a prospective study showing that 54% of children who reported reasonable symptom control had abnormal spirometry [9]. In a recent prospective observational study of children with FEV1 greater than 80% of predicted, the authors demonstrated that low MMEF values as markers of SAD may be present in the group with controlled asthma and appear to be unrelated to the level of asthma control [10]. Kjellberg et al. proved that FEF25-75 detects SAD in 44% of patients, while oscillometry shows SAD in 63%, and multiple breath washout in 54% of children tested [11]. When SAD is defined by oscillometry and/or multiple breath washout abnormality, the percentage increases to 77%. The authors suggest that these methods reflect different aspects of SAD and should be used concurrently. The abnormal small airways parameters in children were not associated with hospitalisations for asthma during the past 12 months [10,12]. The finding would require prospective longitudinal studies to demonstrate the role of SAD in controlling pediatric asthma. These data suggest that monitoring asthmatic patients using subjective symptom control tests alone is not sufficient, as it may underestimate the risk for future severe asthmatic attacks. A recent meta-analysis proved the role of Th2-type inflammation biomarkers, fractional exhaled nitric oxide (FeNO), and eosinophilia in predicting asthma exacerbations [13]. In addition to these parameters, the authors show the predictive role of female gender and increased body mass index in increasing the risk of asthma exacerbation. Analyzing the pulmonary function parameters, it is found that FEV1 post bronchodilator reversibility is correlated with a lower risk for exacerbation [13]. The study included adults with asthma and did not refer to MMEF.

According to a survey conducted in several clinics in Bucharest, Romania, adults show suboptimal control of asthma [14]. Additionally, information about asthma control and the utilisation of spirometry in its assessment remains unknown, mainly due to the absence of systematic studies on representative samples. There is no available data on paediatric asthma. Asthma cases in schoolchildren in Romania are increasing [15]. However, there is a knowledge gap in the literature represented by the lack of data on disease control in the pediatric population in our country. The use of spirometry in children with asthma and the risk of exacerbations are aspects that have not been explored to date. In a previous pilot study conducted by our team we found that FEV1 can be considered a parameter that correlates with the severity of asthma at the time of the spirometry, while MMEF values may be associated more with the progression of the disease [16]. Furthermore, the study we propose also addresses the correlations between MMEF and exacerbations.

The study’s primary objectives were to analyse the quality of spirometry in children and emphasise the importance of accurate spirometry for asthma monitoring. The secondary objective was to evaluate if FEV1 and MMEF values are predictive markers for future exacerbations in children with asthma.

## 2. Materials and Methods

This study is a retrospective observational study conducted in January 2024 in a single tertiary pediatric centre, the Third Pediatric Clinic of the Clinical Hospital for Pediatric Emergencies from Cluj-Napoca, Romania.

### 2.1. Selection of Participants

The study included patients aged between 5 and 18 years, with a confirmed diagnosis of asthma for at least 6 months before joining the study, from rural or urban environments, who performed at least one spirometry test between January 2022 and December 2023. Exclusion criteria were as follows: age under 5 years or over 18 years, exacerbation or use of oral glucocorticoids in the last 7 days, acute respiratory infections, or patients with inconclusive spirometry.

### 2.2. Methods

Technical information. Pulmonary function was assessed using spirometry (BTL-08 Spiro, BTL CardioPoint), which was performed according to the recommendations of the American Thoracic Society (ATS) and the ERS [3] and the Association for Respiratory Technology and Physiology (ARTP)/British Thoracic Society (BTS) guidelines [17]. Participants received instructions to refrain from using any short- or long-acting bronchodilators within 18 h prior to lung function testing. They were also asked to avoid vigorous exercise, smoking/vaping one hour before testing and not wearing clothing that substantially restricts full chest and abdominal expansion. All spirometries were performed in the morning. During spirometry, the child was upright and wore a nose clip. Three technically acceptable spirometry results were obtained, and we analysed the quality of both spirograms. The acceptability criteria used were:

1. For patients aged 10 years or older: an adequate onset of expiration starting from point 0 [expiratory volume ≤ 5% of FVC or 1500 mL, whichever is greater], a rapid rise to peak expiratory flow with a sharp peak, complete expiration (expiratory plateau ≤ 0.025 L in the last second of expiration and an expiratory time ≥ 6 s), and smooth expiration on the flow-volume curve, free from artefacts (such as cough in the first second of forced expiration, non-maximal effort throughout expiration, premature glottis closure, premature termination of expiration, obstruction or air loss around the mouthpiece).
2. For children under 10 years: exhalation over 3 s and absence of coughing or premature glottis closure in the first 0.75 s of exhalation.

The repeatability criteria for children over 10 years old were: the difference between the two highest FVC values must be less than or equal to 0.150 L, and the difference between the two highest FEV1 values must also be less than or equal to 0.150 L. For younger children, the difference between the two highest FVC values must be less than 0.100 L or 10% of the higher value (whichever is greater), and the difference between the two highest FEV1 values must be less than 0.100 L or 10% of the higher value (whichever is greater).

The parameters followed in the study were:

Pulmonary volume and flows: FVC (the volume of gas expelled from the lungs in a forced full exhalation following a maximal inspiration), FEV1, FEV1/FVC ratio, and MMEF. We reported FEV1 and MMEF values as percentages of predictive values and considered normal FEV1 above 80% of predictive value and normal MMEF above 65% of predictive value.

– The level of asthma control according to the GINA guideline, based on symptoms, the number of exacerbations, and lung function: controlled (C), partially controlled (PC), or uncontrolled (UC) asthma.
– The long-term treatment regimen: inhaled corticosteroids, alone or combined with long-acting beta2 adrenergic or long-acting muscarinic antagonist, or leukotriene modifier.

### 2.3. Study Protocol

In a subset of patients, we analysed the presence of exacerbations over a 12-month period and compared the number of these episodes with initial and after-12-month values of both FEV1 and MMEF. This subset of patients was chosen based on the availability of spirometry parameters at baseline and after 12 months.

### 2.4. Statistics

Using EpiInfo’s STATCALC option, the minimum required sample size was calculated. The number of cases needed for a study with 99% power was 319.

Statistical analysis and charting were performed using Microsoft Office Excel 2016 and the Jamovi 2.5 programme. All patient data included in the study were stored in an Excel file.

Patients with controlled, partially controlled, and uncontrolled asthma were analysed separately. Descriptive statistics (mean, 95% confidence interval, number, and proportion) were used to examine the demographic data of the three groups. To highlight the statistical differences between groups for FEV1 and MMEF values, the following statistical tests were employed: the Shapiro–Wilk test for data normality, the Mann–Whitney U test for non-normally distributed data, Fisher’s test for variances in normally distributed data, and Student’s *t*-test for equal variances. Quartiles were calculated to illustrate the statistical significance of quantitative data, while the Chi-Square test was used for qualitative data. The existence of a relationship between spirometry parameters and exacerbation rates was assessed using the Regression function in Data Analysis in Excel. The significance of the correlation between spirometry parameters and the number of exacerbations was quantified by calculating the Spearman Correlation Coefficient for variables that did not follow a normal distribution. The threshold for significance in each test was set at *p* < 0.05.

This study involved a retrospective analysis. Anonymised patient data were handled strictly in compliance with the hospital’s ethical standards and national data protection regulations. The approval from the Clinical Hospital for Pediatric Emergencies, Cluj-Napoca, Romania was obtained on 28 June 2024 (code: 9055). The research was conducted according to the Declaration of Helsinki and approved by the Scientific Research Ethics Committee of “Iuliu Hatieganu” University of Medicine and Pharmacy, Cluj-Napoca, Romania on 10 June 2025 (code: AVZ62). Participants received no financial compensation. General artificial intelligence was not used in this project.

## 3. Results

The study group consisted of 481 children who underwent at least one spirometry test between January 2022 and December 2023. In a subsequent cohort of 88 patients, we monitored spirometry initially and after 12 months, analysing the correlation between the number of exacerbations and the values of FEV1 and MMEF at baseline and after 12 months.

From the initial 481 recorded spirometry manoeuvres, we excluded 65 (13.5%) that did not meet reproducibility criteria. The remaining 416 patients were divided into two groups: children aged 5–10 and patients aged 11–18.

### 3.1. Quality of Spirometry in Children by Age Groups

Spirometry quality was assessed according to the flow-volume and volume-time spirograms as described. The data obtained are presented in Table 1.

In our study, the group aged between 5 and 10 years demonstrated a better initiation of expiration during the spirometry maneuver, although this was not statistically significant. The presence of an expiratory peak and the appropriate duration of expiration were significantly more common in older children (91.3% versus 76.9%; *p* < 0.05; and 42.8% versus 32.2%; *p* < 0.05). In some maneuvers, we observed secondary inspirations during spirometry or a cough.

### 3.2. The Pulmonary Function Tests in Asthma Patients

After excluding inappropriate spirometry tests, we analysed the results of the remaining 276 tests performed on children with asthma. Table 2 summarises the characteristics of the two age groups.

### 3.3. Spirometric Parameters in Children with Asthma According to the Control Level

As expected, most patients with controlled asthma showed normal FEV1 (96.3%) and MMEF (83.8%) values, while 31 patients (43.7%) with uncontrolled asthma had FEV1 values below 80% of the predicted levels. Among children with uncontrolled asthma, MMEF values were below 80% of the predicted levels in 61 cases (85.9%) and under the 65% threshold in 29 patients (40.8%). The FEV1 and MMEF parameter values were compared across the C, PC and UC groups (Table 3).

### 3.4. Long-Term Treatment

The treatment plan for the patients included in the study was based on their level of control, as shown in Table 4. Inhaled corticosteroids (ICS) used were fluticasone propionate or budesonide, administered either as monotherapy or in combination with a leukotriene receptor antagonist (LTRA), such as montelukast, or in fixed combinations with long-acting β2-agonists (LABA), either formoterol or salmeterol, and in severe cases, with long-acting antimuscarinic agents (LAMA), such as tiotropium bromide.

### 3.5. Predictive Value of Spirometry Parameters for Asthma Exacerbation

**Figure 1:**
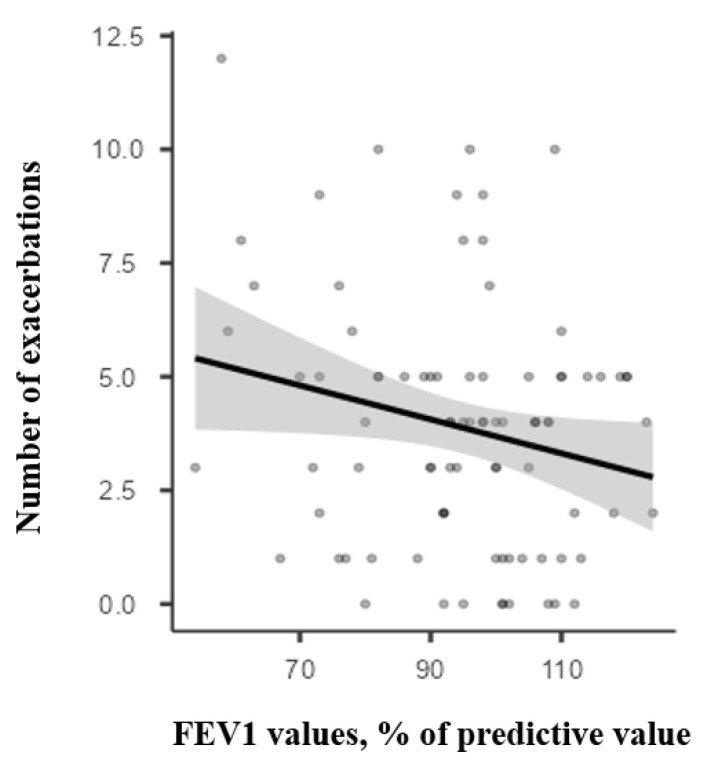
Correlation between the number of exacerbations during 12 months of follow-up and the initial FEV1.

**Figure 2:**
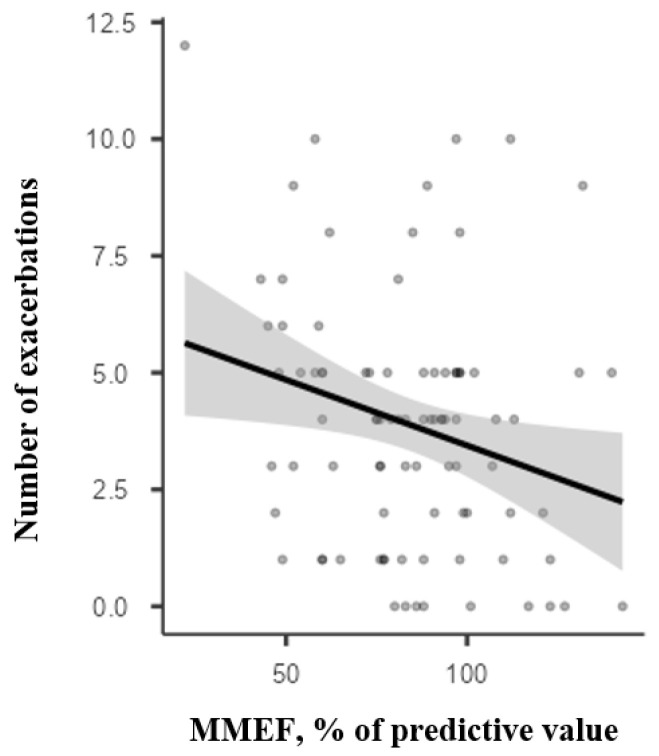
Correlation between the number of exacerbations during 12 months of follow-up and the initial MMEF.

We analysed the clinical progression and pulmonary functional parameters over 12 months in 88 patients. This group comprised 48 children under the age of 10 (29 boys, 60.4%) and 40 children over the age of 11 (23 boys, 57.5%). Most patients had PC asthma (25 patients in the under-10 group and 17 patients in the over-11 group). The group also included children with UC asthma, 9 cases (18.7%) in the younger age group and 8 cases (20%) in the older age group. In these patients, we recorded the number of exacerbations during the 12-month period, the initial spirometry results, and the outcomes at the end of the follow-up. Of these, 79 patients (89.7%) experienced at least one exacerbation during the monitoring period. The number of exacerbations over the 12 months was analysed in relation to spirometry flows. The Spearman correlation coefficient, used for independent non-normally distributed samples, measured the relationship between the two variables. It was weakly negative (Rs = −0.156) and not statistically significant (*p* = 0.146) for initial FEV1 values and the number of exacerbations, and somewhat negatively correlated (Rs = −0.30) with statistical significance (*p* = 0.040) for initial MMEF values. The number of exacerbations according to FEV1 and MMEF values is shown in Figure 1 and Figure 2.

Over the 12-month follow-up period, we observed an improvement in FEV1 values in 38 (43.2%) of our cases, a decline in 47 (53.4%), and similar values at both pulmonary function tests conducted 12 months apart in 3 (3.4%) of our cases. The Wilcoxon matched-pair test proved no statistically significant difference of FEV1 (*p* = 0.473). No correlation was found between the difference in the two FEV1 measurements and the number of exacerbations (Rs = 0.089; *p* = 0.407).

During the 12-month follow-up, we observed an improvement in MMEF values in 47 (53.4%) of our cases, a decrease in MMEF in 38 (43.2%) of our cases, and similar values in 3 (3.4%) of our cases. The difference between initial and final MMEF values were not statistically significant (*p* = 0.29). No correlation was observed between the difference in the two MMEF determinations and the number of exacerbations (RS = 0.101; *p* = 0.339).

Of the nine patients who did not experience exacerbations over one year, three had an FEV1 approximately 19% higher, while three patients experienced an approximately 11% decrease in FEV1 values after 12 months of follow-up. In this group, a mean increase of about 14% (in four patients) and a mean decrease of 23% (in four patients) in MMEF values were observed, with the values remaining unchanged in one patient.

### 3.6. Changes in Long-Term Treatment

After the follow-up period, the treatment regimen was adjusted in 31 of 88 patients (35.2%), either by increasing with new prescribed medications (such as LTRA, LABA, or LAMA), or in 14 (15.9%) cases, by decreasing due to good outcomes, both involving reductions in exacerbations, symptom frequency, and improvements in FEV1 and MMEF. The same long-term treatment was maintained in 43 (48.9%) patients. In our study group, no patient was prescribed medium- or high-dose ICS combined with LABA and/or long-acting antimuscarinic agents (LAMA).

## 4. Discussion

Achieving asthma control and preventing future exacerbations are the main long-term goals in managing children with asthma [18]. In our group, the percentage of uncontrolled asthma was 25.7%. This is slightly higher than the figures reported for children, 25.3% or 22.3% for adolescents in recent years [18]. The percentage of valid spirometry results was lower (66.3%) than in other studies [19]. Children aged 5 to 10 years showed better expiration onset during spirometry manoeuvres than older patients. The presence of an expiratory peak and the appropriate duration of expiration were significantly more common in children over 11 years old.

In our study, we examined the prevalence of SAD and its association with asthma control and the risk of future exacerbations. We analysed 276 (66.3%) tests out of the initial spirometries. Our valid spirometry rate is lower than that reported in other studies with a narrower age range of 6 to 14 years [20]. A success rate for spirometry similar to ours has been noted by other researchers in both patients, at 60%, and in the control group, at 68% [19,21]. When respiratory oscillometry is employed, this percentage increases across all these trials. Pulmonary function test in children require age-appropriate techniques and trained staff [22]. To improve the success rate of spirometry some authors emphasize the significance of a calm and child-friendly testing environment, with playful elements in the testing lab and familiarizing the child with the equipment and procedure [23]. Using age-appropriate language to explain the maneuver and gamification, as well as video tutorials can be helpful. In children aged 5–10 years, the quality of their spirograms was lower compared to those over 11 years old, particularly regarding the presence of expiratory peak and duration of expiration, with statistically significant differences between the age groups (*p* < 0,05). Conversely, younger children performed better in expiration onset and the presence of secondary inspiration during spirometry, although these differences were not statistically significant. This indicates that many spirometers may not be suitable for making definitive diagnoses or for effective monitoring, especially in the high-risk age group.

We compared the three asthma control groups and found a 5.8% and 12.6% difference in FEV1 values (>80% of the predicted) between the C and PC groups, respectively, and between the PC and NC groups. A higher difference was observed in MMEF values (>80% of the predicted): 9.5% and 27.8% between the C and PC groups, respectively, and between the PC and NC. This indicates that MMEF variability is related to symptomatology, whereas FEV1 values show minimal changes regardless of symptoms.

The differences in FEV1 and MMEF values were statistically significant (*p* = 0.0102 and *p* = 0.0001) among the C, PC, and NC groups. Low FEV1 values, below 80% of the predicted, were found in 20.8% of patients in the PC group and 43.7% in the UC group, with similar percentages observed in patients with MMEF values below 65% of the predicted. Values below 80% of the predicted were recorded in 3.7% of patients with controlled asthma for FEV1 and 12.5% for MMEF. This indicates that patients with impaired lung function can be overlooked if only subjective control tests are used without conducting respiratory function tests. For the MMEF parameter, the difference between groups is more evident, with only 14.1% of patients in the UC group having values above 80% of the predicted. This suggests that MMEF values may correlate better with symptoms and airway changes. Therefore, performing spirometry at every routine check-up is crucial to identify patients with low MMEF values and no obvious symptoms. This allows for appropriate therapeutic decisions to be made, reducing asthma morbidity and mortality.

The FEV1 is currently used to classify airflow obstruction and its severity, as it primarily reflects the function of the larger airways. A normal FEV1 value measured in the morning is a key criterion for controlled asthma. In patients with low FEV1, it is necessary to increase background treatment even if no symptoms are present. The most commonly used parameter for SAD is MMEF [5,24]. Other psychological tests may also characterise SAD [25]. Most studies indicate that SAD is more prevalent in patients with uncontrolled asthma [26,27]. However, the usefulness of MMEF in assessing the risk of exacerbation and guiding clinical decisions remains debated. The MMEF shows some technique-dependent variability, which reduces its reliability as an independent diagnostic tool and limits its usefulness in standard clinical practice, particularly in specific age groups [5,28].

The assessment of risk for exacerbation has become an essential part of the follow-up plan for these patients. Besides common triggers, environmental, psychological, and social factors are recognised. Key tools include pulmonary function tests, mainly spirometry and oscillometry. For both diagnosis and monitoring, extensive literature has demonstrated the role of FEV1. Recent studies emphasise the important role of the FEV1/FVC ratio or preserved ratio impaired spirometry (PRISm) in monitoring patients of all ages with obstructive pulmonary disorder [29]. PRISm is associated with higher mortality and more frequent respiratory symptoms in adults.

Our study found no link between FEV1 and the risk of exacerbations. This lack of predictability of the FEV1 parameter for disease progression was also observed in a 2016 study, where low FEV1 could not be linked to the risk of future exacerbations in children, but only in adults [30]. This phenomenon may be related to children with asthma not exhibiting significant airway remodelling with a permanent decrease in FEV1 values, which can occur in adult patients with asthma [31]. Another study found no association between symptom severity, treatment intensity, and predicted FEV1 values [32]. This may be explained by the fact that FEV1 and peak expiratory flow are less sensitive in detecting bronchial obstruction than small airway parameters (mean expiratory flow rate at 75% of vital capacity, MEF75; mean expiratory flow rate at 50% of vital capacity, MEF50; the mean expiratory flow rate at 25% of vital capacity, MEF25; and MMEF in children with asthma. In a recent study where the classification criterion for asthma control was ACT, the authors showed that in patients with uncontrolled asthma, FEV1 was normal in most patients [33]. However, MEF50 and MEF25 were low in these patients, and their reversibility correlated with that of FEV1. When comparing the two parameters reflecting SAD, MEF25 had higher specificity and better predictive value. The recent meta-analysis by Meulmeester FL shows that FEV1 post-bronchodilator reversibility has a non-linear relationship with future severe exacerbations [13].

In this study, 85.9% of our patients with UC asthma had MMEF below 80% of the predicted value, and 40.8% had MMEF below 65%, compared to the C group, where 12.5% and 3.7% respectively had low MMEF values. For FEV1, 3.7% of children in the C group, 20.8% in the PC group, and 43.7% in the UC group showed values below 80% of the predicted. Other studies have also reported similar findings [11]. Some authors suggest that FEV1 can be considered a parameter that correlates with asthma severity during spirometry, and that MMEF may be associated with disease progression. Currently, there is data indicating that in patients with normal FEV1 and uncontrolled asthma, MMEF is a much more sensitive parameter [11].

The correlation between exacerbation rate and MMEF values over 12 months was weak for a Spearman correlation coefficient of −0.30 and was statistically significant. This indicates a potential link between MMEF values and the risk of future exacerbations. A 2015 study [12] associated small airway function with asthma symptoms and exacerbation risk. More recently, Lazova S. showed that patients with small airway obstruction more often experience symptoms related to physical exertion, allergen contact, and climate changes regardless of FEV1 values [34]. Other researchers identified a small group of asthmatic children with normal FEV1 and low MMEF [35]. These patients are at greater risk of adverse reactions, such as exacerbations, hospitalisations, and progressive decline in respiratory function.

Spirometry indicates small airway involvement, with MMEF as a typical marker [36]. A recent study evaluated the risk of severe exacerbation in 154 children followed over three months [37]. Of these, 82.4% of the spirometry tests were valid. Among them, 7.4% experienced severe exacerbations, 18.3% visited the emergency room, and 11.5% needed an increase in treatment steps [37]. The authors found that the forced mid-expiratory flow, FEF25-75, had a greater predictive ability for exacerbation risk than other parameters. Low FEF25-75 values do not specifically correlate with the air trapping phenotype but are useful for predicting airflow limitation and small airway disease [37].

In our study, MMEF and FEV1 values decreased in 53.4% of patients who experienced exacerbations during the 12 months. In 66.7% of patients who remained free of exacerbations at the 12-month mark, FEV1 increased by 8.7% from baseline. Among the patients without exacerbations over 12 months, 50% had higher MMEF values by approximately 10.2% compared to their initial values.

In a recent study, the authors emphasise the importance of using multiple parameters to assess the progression of children with asthma [38]. They propose a multidimensional approach that includes FENO, ACT, and the GINA Asthma Control Questionnaire, alongside clinical data and spirometry. These measures have been shown to help determine the degree of control. The authors demonstrated that FENO correlates more closely with control level than spirometry and oscillometry [38,39]. This suggests that lung function does not necessarily correspond with clinical progression and symptom management. Respiratory oscillometry was recently added to the monitoring plans for children with asthma, but it is still not widely accessible. It offers several important advantages over spirometry, mainly requiring minimal cooperation from the patient and providing higher sensitivity in assessing SAD [36]. The usefulness of this method has increased as recent evidence indicates it is suitable for predicting asthma exacerbations [36]. Respiratory oscillometry is also recommended for assessing adult lung function. Recent data show that it is reliable in diagnosing SAD, which correlates with the risk of asthma exacerbations [24,37].

The European Respiratory Society guidelines do not support the use of MMEF in the diagnosis of asthma, but rather as a complementary test in conjunction to spirometry and clinical symptoms and emphasize its utility in identifying poor asthma outcome [40]. In children, reduced MMEF can indicate small airways disease with increased risk of exacerbations and hospitalization even if FEV1 is normal, suggesting the need for step-up treatment [34].

Our results showed certain relationship between MMEF and the risk of asthma exacerbation. In our study group, clinical evolution and lung function parameters required long-term treatment adjustment in 35.2% of patients after 12 months of monitoring. Our study group did not include patients on chronic oral corticosteroids or biologic therapy.

### Limitations of the Study

The study was designed to evaluate the individual performance of spirometry, and we did not investigate its utility in multivariate models for predicting exacerbations. The aim was to assess the difference between FEV1 and MMEF regarding long-term progression.

The main limitation of this study is the small number of asthma patients included in the one-year follow-up analysis. We excluded from the analyzed group patients who received corticosteroid therapy 7 days prior to spirometry. Some authors believe that this medication could affect SAD over a longer period of time. In fact, in our group, no patient received systemic corticosteroid therapy in the previous 4 weeks, although this was not the exclusion criterion. ACT is a useful tool for assessing asthma control. Some studies have shown a lack of correlation between lung function and ACT [30]. In our study, we did not use ACT. We also did not include the bronchodilator response of spirometric parameters in the analysis, nor did we perform a stratified analysis of respiratory functional phenotypes in the patient group.

A prospective study, with a well-established protocol, involving a larger and more representative national sample, could offer a far more comprehensive view of the relationship between asthma symptoms and spirometric values, as well as their true significance in predicting the disease’s progression. To better associate minor airway impairment with the risk of exacerbations and a negative asthma trajectory, more sensitive methods for assessing SAD are required. The oscillometric method or the Multiple Breath Washout technique are more practical for such assessments. According to a recent study [34], both methods provide substantial information for distinguishing asthmatic patients with normal spirometry from non-asthmatic patients.

## 5. Conclusions

Pulmonary function tests in children are still a challenge, but of great importance for the diagnostic and management of asthma. This study showed a reduced percentage of valid spirometry tests in children, emphasizing the need for age-specific tests and appropriate environment conditions.

Spirometry, especially FEV1, is indicated for asthma diagnosis and monitoring. Besides this, MMEF is a marker of small airways disease but did not demonstrate diagnostic utility because of its high variability and poor reproducibility.

MMEF remains a complementary parameter in the management of asthma along with FEV1 and clinical features. In children, MMEF can be used as a marker of poor outcome, even if FEV1 is normal. Our results suggest that evaluating MMEF alongside FEV1 in children with asthma, could contribute to better identification of the risk of exacerbation.

Specialized diagnostic tools for small airways disease may identify more accurately different levels of asthma control and could represent future directions of the research in this field.

## Figures and Tables

**Table 1:** Spirometry quality assessment by age group.

	Patients Aged 5–10 Years (N = 208)	Patients Aged 11–18 Years (N = 208)	*p*
Correct expiration onset, no (%)	150 (72.1%)	137 (66.9%)	0.168
Presence of expiratory peak, no (%)	160 (76.9%)	190 (91.3%)	<0.05
Age-appropriate exhalation time *, no (%)	67 (32.2%)	89 (42.8%)	0.025
Presence of secondary inspiration, no (%)	12 (5.8%)	16 (7.7%)	0.433

* The required exhalation time in children aged 5–10 is 3 s, and in children over 11, it is 6 s.

**Table 2:** Baseline characteristics of the participants.

	Patients Aged 5–10 Years	Patients Aged 11–18 Years
Number of cases	157	119
Sex, n (%)		
- Female	61 (38.9)	56 (47.1)
- Male	96 (61.2)	63 (52.9)
Age, years, mean (±SD)	7.76 (±1.8)	14.4 (±2.1)
Controlled asthma, n (%)	43 (27.4)	37 (31.1)
Partially controlled, n (%)	78 (49.6)	47 (39.5)
Uncontrolled, n (%)	36 (22.9)	35 (29.4)
Controlled asthma		
- FEV1 *, mean (±SD)	99.5 (±11.0)	99.5 (±11.8)
- FEV1 < 80% of pred, n (%)	2 (4.6)	1 (2.7)
- FEV1/FVC, mean (±SD)	103 (±7.2)	103 (±7.68)
- MMEF *, mean (±SD)	101 (±16.6)	100 (±20.7)
- MMEF < 80% of pred, n (%)	4 (9.3)	6 (16.2)
- MMEF < 65% of pred, n (%)	2 (4.7)	1 (2.7)
Partially controlled		
- FEV1 *, mean (± SD)	93.8 (±15.7)	93 (±14.8)
- FEV1 < 80% of pred, n (%)	18 (23)	8 (17.0)
- FEV1/FVC, mean (±SD)	99.3 (±9.49)	98.4 (±8.9)
- MMEF *, mean (±SD)	90.5 (±27.4)	89.3 (±23.4)
- MMEF < 80% of pred, n (%)	32 (41)	11 (23.4)
- MMEF < 65% of pred, n (%)	10 (12.8)	8 (17.0)
Uncontrolled		
- FEV1 *, mean (± SD)	82.9 (±17.6)	78.8 (±12.5)
- FEV1 < 80% of pred, n (%)	17 (47.2)	14 (40)
- FEV1/FVC, mean (±SD)	91.4 (±13.3)	88.7 (±8.1)
- MMEF *, mean (±SD)	66.1 (±21.8)	61.3 (±16.2)
- MMEF < 80% of pred, n (%)	30 (83.3)	31 (88.6)
- MMEF < 65% of pred, n (%)	11 (30.6)	18 (51.4)

* % of predictive values.

**Table 3:** Comparison between FEV1 and MMEF values for C, PC and UC groups.

	C (n = 80)	PC (n = 125)	UC (n = 71)	*p*
FEV1 *, mean (±SD)	99.3 (±11.2)	93.5 (±15.3)	80.9 (±15.4)	0.0102
MMEF *, mean (±SD)	101 (±18.5)	91.5 (±27.3)	63.7 (±19.3)	0.0001

* % of predictive values.

**Table 4:** Step of treatment at the inclusion in the study.

	C (n = 80)	PC (n = 125)	UC (n = 71)
No maintenance treatment, n (%)	49 (61.3)	24 (19.2)	0
Monotherapy (low-dose ICS or LTRA), n (%)	30 (37.5)	87 (69.6)	5 (7.0)
Medium-dose ICS or combination (low-dose ICS + LTRA/LABA), n (%)	1 (1.3)	11 (8.8)	26 (36.6)
High-dose ICS (±LABA and/or LTRA) or medium-dose ICS + LABA, n (%)	0	3 (2.4)	40 (56.3)

ICS—inhaled corticosteroids; LTRA—leukotriene receptor antagonist; LABA—long-acting β2-agonists.

## Data Availability

The original contributions presented in this study are included in the article. Further inquiries can be directed to the corresponding author.

## Abbreviations

The following abbreviations are used in this manuscript:

ACT	Asthma Control Test
ARTP	Association for Respiratory Technology and Physiology
ATS	American Thoracic Society
BTS	British Thoracic Society
C	Controlled (asthma group)
ERS	European Respiratory Society
FEF25-75	Forced Expiratory Flow between 25 and 75% of FVC
FEF50	Forced Expiratory Flow at 50% of FVC
FEV1	Forced Expired Volume in 1 s
FeNO	Fractional Exhaled Nitric Oxide
FVC	Forced Vital Capacity
ICS	inhaled corticosteroids
LABA	Long-acting β2-Agonists
LAMA	Long-Acting Muscarinic Antagonists
LTRA	Leukotriene Receptor Antagonist
MEF25	Mean Expiratory Flow rate at 25% of vital capacity
MEF50	Mean Expiratory Flow rate at 50% of vital capacity
MMEF	Maximal Mid-Expiratory Flow
PC	Partially Controlled asthma group
SAD	Small Airway Dysfunction
SD	Standard Deviation
UC	Uncontrolled asthma group
